# MicroRNA Profiling of Bone Marrow Plasma Extracellular Vesicles in Multiple Myeloma, Extramedullary Disease, and Plasma Cell Leukemia

**DOI:** 10.1002/hon.70036

**Published:** 2025-01-13

**Authors:** Jana Gregorova, Monika Vlachova, Petra Vychytilova‐Faltejskova, Adela Dostalova, Tereza Ruzickova, Marek Vecera, Lenka Radova, Vendula Pospichalova, Stanislava Sladecek, Martina Hyzdalova, Jana Kotaskova, Marie Jarosova, Josef Masek, Klara Benesova, Jiri Jarkovsky, Lucie Rihova, Renata Bezdekova, Martina Almasi, Ivanna Boichuk, Martin Stork, Ludek Pour, Sabina Sevcikova

**Affiliations:** ^1^ Babak Myeloma Group Department of Pathophysiology Faculty of Medicine Masaryk University Brno Czech Republic; ^2^ Centre for Molecular Medicine Central European Institute of Technology Masaryk University Brno Czech Republic; ^3^ Department of Experimental Biology Faculty of Science Masaryk University Brno Czech Republic; ^4^ Department of Pharmacology and Toxicology Veterinary Research Institute Brno Czech Republic; ^5^ Department of Internal Medicine Hematology and Oncology University Hospital Brno Brno Czech Republic; ^6^ Faculty of Medicine Institute of Biostatistics and Analyses Masaryk University Brno Czech Republic; ^7^ Department of Clinical Hematology University Hospital Brno Brno Czech Republic

**Keywords:** extramedullary disease, microRNAs, multiple myeloma, plasma cell leukemia, small extracellular vesicles, small RNA sequencing

## Abstract

Multiple myeloma is a plasma cell malignancy characterized by an abnormal increase in monoclonal immunoglobulins. Despite significant advances in treatment, some patients progress to more aggressive forms of multiple myeloma, including extramedullary disease or plasma cell leukemia. Although the exact molecular mechanisms are not known, several studies have confirmed the involvement of small extracellular vesicle‐enriched microRNAs in multiple myeloma progression. Therefore, we performed expression profiling of these molecules in bone marrow plasma of multiple myeloma, extramedullary disease, and plasma cell leukemia patients using small RNA sequencing to identify novel molecules involved in disease pathogenesis. In total, 42 microRNAs were significantly dysregulated among analyzed subgroups. Independent validation by RT‐qPCR confirmed elevated levels of miR‐140‐3p, miR‐584‐5p, miR‐191‐5p, and miR‐143‐3p in multiple myeloma patients compared to extramedullary disease and plasma cell leukemia patients. Subsequent statistical analysis revealed significant correlations between patient clinical characteristics or flow cytometry parameters and microRNA expression. These results indicate that dysregulation of microRNAs could contribute to multiple myeloma progression.

## 1 Introduction

Multiple myeloma (MM) is the second most common hematological malignancy, characterized by monoclonal immunoglobulin in serum and/or urine of patients produced by malignant plasma cells (PCs) present in the bone marrow (BM) [1]. Survival of MM cells is dependent on the BM microenvironment [2].

MM may progress into extramedullary disease (EMD) or plasma cell leukemia (PCL). EMD is an aggressive form of MM characterized by a subclone of PCs migrating out of the BM niche; this subclone infiltrates soft tissues, sometimes forming tumors [3, 4, 5, 6, 7]. PCL is a rare, aggressive form of monoclonal gammopathies characterized by more than 5% of circulating PCs in peripheral blood [8, 9].

The BM microenvironment plays a crucial role in MM progression [5]. Thus, understanding the dynamic crosstalk between microenvironment components and malignant cells is essential to uncovering the mechanisms of disease formation and progression. The role of extracellular vesicles (EVs) in immune signaling was first highlighted by Raposo et al. [10], who demonstrated that EVs secreted by B lymphocytes carry MHC II class molecules and can trigger antigen‐specific immune responses. EVs are membrane‐enclosed particles that can be classified based on biogenesis, size, and mode of cellular release, such as exosomes, ectosomes, or apoptotic bodies [11]. They are produced by all cells and are involved in physiological and pathological processes, including cancer; they contain a spectrum of bioactive molecules: nucleic acids, proteins, lipids, and different metabolites [12, 13]. Small EVs refer to vesicles of < 200 nm in diameter, including exosomes [11]. Actively released by cancer cells into peripheral blood, small EVs represent a promising source of minimally invasive biomarkers for early diagnosis or therapy response monitoring [14].

MicroRNA (miRNA) is a bioactive molecule found in small EVs. MiRNAs are small non‐coding RNAs that post‐transcriptionally regulate gene expression and function as modulators of cellular processes, including remodeling the BM microenvironment [4, 15, 16, 17]. They are released into circulation by various mechanisms, such as passive leakage from cells suffering injury or inflammation, during apoptosis or necrosis of cells, via active secretion as protein‐miRNA complexes, or enclosed in EVs [18]. Several studies showed the possible influence of small EVs miRNAs on MM progression [19, 20, 21, 22, 23].

We performed small RNA sequencing (RNA‐seq) of miRNAs isolated from small EVs in BM plasma samples of MM, EMD, and PCL patients to characterize miRNA profiles in the BM microenvironment.

## 2 Materials and Methods

### 2.1 Patients' Characteristics

In total, 76 patients (29 MM, 34 EMD, and 13 PCL) diagnosed at the University Hospital Brno, Czech Republic, between 2008 and 2020 were involved in the study. All samples were collected at the time of diagnosis. The diagnosis of EMD was based on imaging techniques (PET‐CT, MRI, low dose CT) and was confirmed by histopathological examination of biopsied samples. PCL diagnosis was set at 5% of circulating PCs [24, 25]. Clinical characteristics, therapy, and treatment data were collected from the Registry of Monoclonal Gammopathies (RMG) (summarized in Supporting Information S2: Table S1). All patients included in this study signed an informed consent form approved by the hospital's Ethics committee in accordance with the current Declaration of Helsinki.

### 2.2 Sample Preparation

BM plasma samples were frozen at −80°C as 0.5 mL aliquots. Each aliquot was thawed only once [5].

### 2.3 Interphase Fluorescent in‐situ Hybridization

Interphase fluorescent in situ hybridization (I‐FISH) analysis of common MM aberrations was performed on separated PCs as described [26]. The positivity cut‐off was set according to the European Myeloma Network recommendations [27].

### 2.4 Flow Cytometry Analysis of Phenotype and Clonality of Bone Marrow Plasma Cells

Samples were incubated with fluorescently labeled monoclonal antibodies (MoAbs) (Supporting Information S2: Table S2) for surface phenotyping and clonality assessment using BD FACSCanto II (Becton Dickinson, United States of America) as described [4]. Surface expression on clonal PCs was evaluated according to the European Myeloma Network [4, 9, 28, 29].

### 2.5 Isolation of Small Extracellular Vesicles

BM plasma aliquots were centrifuged at 1200 × g for 10 min at 4°C to remove cell debris. Then, 500 μL of each sample was passed through qEVoriginal Size Exclusion Columns (35 nm; Izon Science, UK) to isolate small EVs. Fractions were analyzed by multi‐angle dynamic light scattering (MADLS) and western blotting (WB). Optimal fractions (8 and 9, 500 μL each) were combined and concentrated to 200 μL using Amicon Ultra 4 mL filter (Merck Millipore, United States of America) for RNA isolation. For quality control, 500 μL of BM plasma from 2 MM, 2 EMD, and 2 PCL samples were processed and analyzed by cryo‐EM, MADLS, and WB.

### 2.6 Multi‐Angle Dynamic Light Scattering

Approximately 50 μL of the EV suspension was placed in low‐volume quartz batch cuvette ZEN2112 (Malvern Pananalytical Ltd, Malvern, UK) and measured using multi‐angled dynamic light scattering technique (MADLS) on Zetasizer Ultra (Malvern Pananalytical Ltd) at constant temperature 25°C as described [30]. The device was equipped with HeNe Laser (633 nm) and three detectors at the following angles: 173° (backscatter), 90° (side scatter), and 13° (forward scatter). The measured data were evaluated using ZS Xplorer software version 1.50 (Malvern Pananalytical Ltd). The measured values included hydrodynamic size, polydispersity index (PdI), and concentrations of EVs; the results are reported as mean value (*n* = 6) ± standard deviation.

### 2.7 Cryo‐Electron Microscopy

Samples for cryo‐EM were vitrified using FEI Vitrobot Mark IV as described [30]. 4 μL of the sample was applied on fresh plasma‐cleaned Cu (Quantifoil R 2/1300 mesh) grids; vitrification in liquid ethane was performed with the following setting: blot force −1; blot time 6 s, wait time 45 s; 100% humidity at 4°C. The grids were subsequently mounted to the Autogrid cartridges and imaged on Talos Glacios (Thermo Fisher Scientific) cryo TEM microscope operated at 200 kV using Falcon4I direct electron detection camera at XYx (for overview) or XYx (for detail) nominal magnification with the underfocus 2 μm and the overall dose of < 30 e/Å2.

### 2.8 SDS‐PAGE and Western Blotting

Protein sample lysates were prepared by direct lysis of EVs in 5× concentrated reducing Laemmli buffer. Lysates were separated according to their molecular mass on 15% SDS‐PAGE and transferred to Immobilon‐P Membrane (Millipore) as described [30]. SDS‐page and WB were performed as described [30]. The following antibodies were used: rabbit antiFlotillin1 (A3023, Exbio, dilution 1:500), mouse anti‐Flotillin2 (BD610383, BD, dilution 1:500), rabbit anti‐CD9 (A19027, ABclonal, dilution 1:500), rabbit anti‐ApoA‐I (sc‐30089, Santa Cruz Biotechnology, dilution 1:1000).

### 2.9 Isolation of RNA From Small Extracellular Vesicles

Total RNA enriched for small RNA (including miRNA) was isolated from small EVs using miRNeasy Micro Kit (Qiagen, Germany) and assessed using NanoDrop ND‐1000 and Qubit RNA HS Assay (Thermo Fisher Scientific, United States of America) as described [31].

### 2.10 Small RNA Sequencing

8 MM samples, 6 EMD, and 8 PCL were used for small RNA‐seq. Libraries were prepared and assessed as previously described [4]; adapter dimers were trimmed by the Pippin Prep system (Sage Science, United States of America). Equimolar amounts of libraries were pooled at a final concentration of 2 nmol·l^−1^ and concentrated to 30 μL. Sequencing was performed using the NextSeq 500 Reagent kit v2 (Illumina, United States of America).

### 2.11 RT‐qPCR Validation of Small RNA Sequencing

For validation, 64 samples were analyzed: 24 MM samples, 32 EMD samples, and 8 PCL samples. 3 MM, 4 EMD, and 3 PCL samples underwent RNA‐seq as well. RT‐qPCR was performed as described previously with 1 ng of input RNA [31]. The miRNA assay IDs are listed in Supporting Information S2: Table S3.

### 2.12 Bioinformatics and Statistical Analyses

Count‐based miRNA expression data from fastq files were generated by the Chimira tool. All sequences were adapter trimmed and mapped against miRBase v22, allowing up to 2 mismatches per sequence. Further analyses were performed using R/Bioconductor packages. MiRNAs with less than 20 reads in the sum of all samples were dropped. The read counts were pre‐normalized by adding normalization factors within the edgeR package [32] and further between‐sample normalized by the voom function in the LIMMA package [33, 34]. After the normalized expression levels were determined, the differentially expressed miRNAs among MM, EMD, and PCL samples were screened by applying linear model fitting and the Bayes approach. The obtained *p*‐values were adjusted for multiple testing using the Benjamini–Hochberg method.

RT‐qPCR data were analyzed as described. The average expression levels of miRNAs were normalized using miR‐92a‐3p, the most stable miRNA based on small RNA‐seq analysis, as verified by the geNorm algorithm [35].

Differences among groups of patients were tested using Fisher's exact test and Kruskal‐Wallis test. Correlation in continuous parameters was assessed using Spearman's correlation coefficient. OS (overall survival) and PFS (progression‐free survival) from the time of MM, EMD, or PCL diagnosis were plotted using Kaplan Meier method. The log‐rank test estimated the statistical significance of the difference between the curves. The Cox proportional hazards model was performed to explore the univariate association of risk factors with OS and PFS. *p*‐values less than 0.05 were considered statistically significant (all tests were two‐sided). Analysis was performed using SPSS software (IBM SPSS Statistics for Windows, Version 28.0.).

## 3 Results

### 3.1 Small RNA Sequencing of microRNAs From Small Extracellular Vesicles

We isolated small EVs from BM plasma samples and characterized them according to MISEV2023 guidelines (Supporting Information S1: Figure S1).

The next part of the study was performed as a two‐step biomarker study: small RNA‐seq was performed, followed by RT‐qPCR.

**FIGURE 1:**
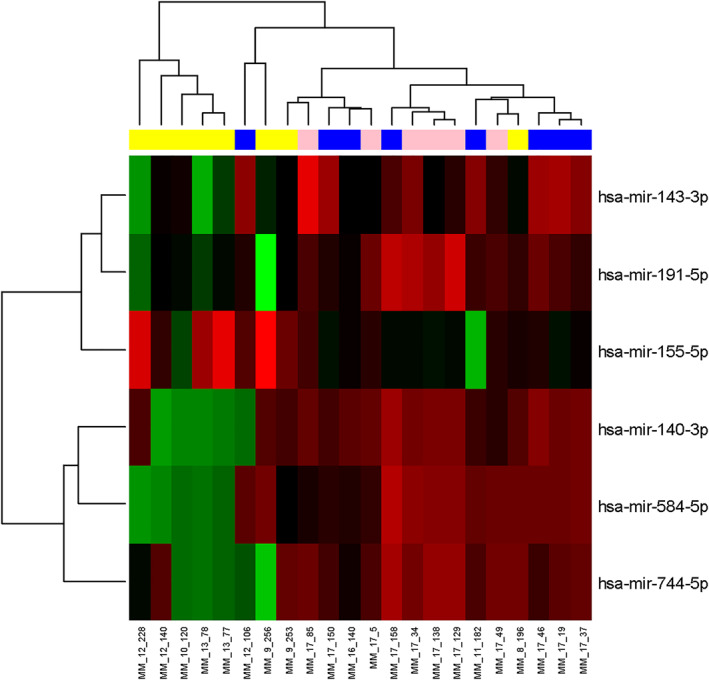
Clustrogram and heatmap visualizing 6 miRNAs differentially expressed among 8 multiple myeloma patients (MM, blue), 6 extramedullary disease patients (EMD, pink), and 8 plasma cell leukemia patients (PCL, yellow) (adjusted *p* ≤ 0.1) in the exploration phase of the study. Red represents high expression, and green represents low expression.

Small RNA‐seq was performed on 8 MM, 6 EMD, and 8 PCL patients, identifying 1128 miRNAs. Further analysis showed 222 miRNAs with more than one read per million in at least 8 samples. In total, 42 different miRNAs were found to be significantly dysregulated among analyzed subgroups (*p* < 0.05); 6 miRNAs (miR‐584‐5p, miR‐191‐5p, miR‐744‐5p, miR‐143‐3p, miR‐155‐5p, and miR‐140‐3p) were chosen for validation in the second step of the study based on log FC and *p*‐value (Figure 1, Table 1).

**TABLE 1:** The 6 most dysregulated miRNAs identified in the exploration phase of the study.

miRNA	logFC	logFC	logFC	*p*‐value	Adjusted
	EMD versus MM	PCL versus MM	PCL versus EMD		*p*‐value
miR‐584‐5p	−0.379	−5.278	−4.898	0.00015	0.03348
miR‐191‐5p	0.409	−1.640	−2.049	0.00037	0.04136
miR‐744‐5p	1.321	−3.338	−4.659	0.00167	0.08129
miR‐143‐3p	−0.055	−1.516	−1.461	0.00182	0.08129
miR‐155‐5p	0.616	2.154	1.538	0.00183	0.08129
miR‐140‐3p	0.403	−3.837	−4.241	0.00255	0.09433

Abbreviations: EMD, extramedullary disease; FC, fold change; MM, multiple myeloma; PCL, plasma cell leukemia.

### 3.2 Validation of microRNAs Dysregulated Among the Diagnostic Subgroups

**FIGURE 2:**
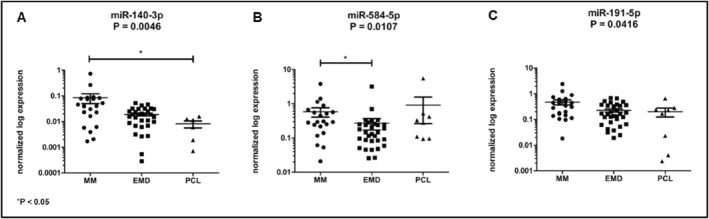
Significantly dysregulated miRNAs among analyzed subgroups of patients. (A) miR‐140‐3p is overexpressed in MM patients compared to PCL patients (*P* = 0.0132). (B) miR‐584‐5p is significantly increased in MM patients compared to EMD patients (*P* = 0.0049). (C) miR‐191‐5p is dysregulated among analyzed subgroups (*P* = 0.0416).

Six miRNAs were selected for the validation phase of the study. Based on the results of the Kruskal‐Wallis test, significant differences in expression were observed in the case of miR‐140‐3p (*p* = 0.0046, Figure 2A), miR‐584‐5p (*p* = 0.0107, Figure 2B), and miR‐191‐5p (*p* = 0.0416, Figure 2C). Table 2 summarizes the results from the statistical analysis of the normalized expression values.

**TABLE 2:** RT‐qPCR validation of miRNAs dysregulated among the three diagnostic subgroups.

miRNA	MM	EMD	PCL	*p*‐value
	Median	Median	Median	
	(min‐max)	(min‐max)	(min‐max)	
miR‐155‐5p	0.06 (0.01–0.91)	0.06 (0.01–1.81)	0.08 (0.00–2.13)	< 0.99
miR‐140‐3p	0.04 (0.00–0.73)	0.02 (0.00–0.05)	0.01 (0.00–0.02)	**0.0046**
miR‐584‐5p	0.29 (0.02–3.81)	0.14 (0.03–3.16)	0.30 (0.09–5.46)	**0.0107**
miR‐191‐5p	0.34 (0.02–2.40)	0.16 (0.02–0.68)	0.20 (0.00–0.66)	**0.0416**
miR‐143‐3p	0.15 (0.01–3.40)	0.07 (0.01–0.60)	0.08 (0.01–1.30)	0.0502
miR‐744‐5p	0.08 (0.00–0.34)	0.04 (0.00–0.23)	0.05 (0.01–0.07)	0.1733

*Note:* Additionally, downregulation of miR‐143‐3p in EMD compared to MM patients was observed (*p* = 0.0158). The expression levels of miR‐744‐5p did not differ among the analyzed subgroups (*p* = 0.1733). Bold values indicate statistically significant.

Abbreviations: EMD, extramedullary disease; MM, multiple myeloma; PCL, plasma cell leukemia.

**FIGURE 3:**
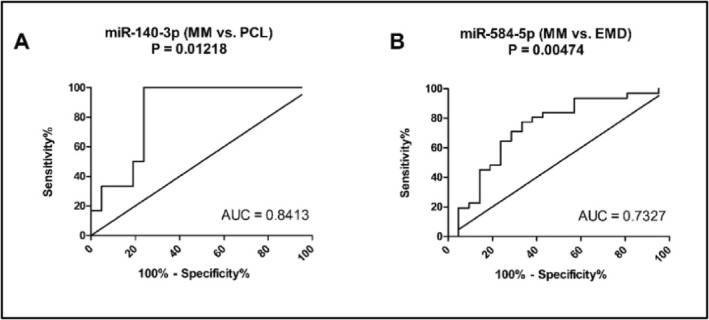
Receiver operating characteristic (ROC) analysis for significantly dysregulated miRNAs. (A) miR‐140‐3p distinguishes between MM and PCL patients with a sensitivity of 100% and specificity of 76% (AUC = 0.8413; cut‐off value: 0.01604). (B) miR‐584‐5p distinguishes between MM and EMD patients with a sensitivity of 77% and specificity of 67% (AUC = 0.7327; cut‐off value: 0.2524).

Based on the results of ROC analysis, miR‐140‐3p (AUC = 0.8413, sensitivity 100%, specificity 76%, cut‐off: 0.01604; Figure 3A) distinguished MM and PCL patients with high sensitivity and specificity, while satisfactory differentiation between MM and EMD patients was based on the expression of miR‐584‐5p (AUC = 0.7327, sensitivity 77%, specificity 67%, cut‐off: 0.2524; Figure 3B).

### 3.3 Differences in Clinical, Cytogenetic, and Flow Cytometric Parameters of Patients

Only 44 patients had evaluable I‐FISH results. Statistically significant differences were observed for *IGH* disruption (positive rate 17.6% in MM, 41.2% in EMD, and 80.0% in PCL; *p =* 0.002), and t (4; 14) translocation (positive rate 0.0% in MM, 5.9% in EMD, and 40% in PCL; *p =* 0.012). Other cytogenetic aberrations were not statistically significant (summarized in Supporting Information S2: Table S4).

Flow cytometry showed differences in PCs according to expression of CD19, CD28, CD117, CD200, and in number of clonal PC among the analyzed groups (summarized in Supporting Information S2: Table S5).

The median length of follow‐up was 20.8 months (range: 0.3–67.9) in MM, 14.4 months (range: 0.0–56.2) in EMD, and 2.0 months (range: 0.2–69.5) in PCL patients. Median PFS was 12.6 months (95% CI: 9.5–15.7) in MM, 9.3 months (95% CI: 6.4–12.2) in EMD, and 1.0 months (95% CI: 0.6–1.3) in PCL (Supporting Information S1: Figure S2A). Median OS was 34.9 months (95% CI: 23.7–46.0) in MM, 32.3 months (95% CI: 12.1–52.5) in EMD, and 2.0 months (95% CI: 0.0–7.6) in PCL (Supporting Information S1: Figure S2B).

### 3.4 Correlation of miRNA Expression Levels With Clinical and Flowcytometric Data

Several significant correlations were found between miRNA levels and clinical characteristics. Elevated levels of LDH (lactate dehydrogenase) were observed in patients with downregulation of miR‐143‐3p, miR‐140‐3p, miR‐744‐5p, and miR‐191‐5p (Supporting Information S2: Table S6). The correlation of miRNA quantity with categorical clinical characteristics of patients at diagnosis did not show any significant correlation apart from M‐protein type and miR‐140‐3p (Supporting Information S2: Table S6).

Analysis of miRNA quantity and flow cytometric parameters revealed a positive correlation between CD138^+^CD38^+^ PCs and miR‐584‐5p; a similar association was observed for miR‐191‐5p. Further, expression of miR‐584‐5p positively correlated with clonal PCs. Positive correlations were found for miR‐191‐5p and CD19^+^ PCs, as well as for miR‐744‐5p and CD117^+^ PCs. An increase in nestin^+^ PCs was observed in connection to the downregulation of miR‐191‐5p and miR‐744‐5p (Table 3).

**TABLE 3:** Correlation of miRNA quantity and flow cytometric parameters.

miRNA	Flow cytometric parameter	N	*r*_S_	*p*‐value^a^
miR‐140‐3p	CD20^+^ PCs	61	0.247	0.055
miR‐584‐5p	CD138^+^CD38^+^ PCs	64	0.257	**0.040**
	CD117^+^ PCs	57	0.230	0.086
	Clonal PCs	64	0.280	**0.025**
miR‐191‐5p	CD138^+^CD38^+^ PCs	64	0.269	**0.032**
	CD19^+^ PCs	61	0.288	**0.024**
	CD81^+^ PCs	55	−0.240	0.078
	CD117^+^ PCs	57	0.248	0.063
	nestin^+^ PCs	53	−0.281	**0.041**
miR‐143‐3p	CD138^+^CD38^+^ PCs	64	0.232	0.065
	CD200 PCs	46	0.248	0.096
miR‐744‐5p	CD117^+^ PCs	57	0.294	**0.026**
	nestin^+^ PCs	53	−0.366	**0.007**

*Note:* Bold values indicate statistically significant.

^a^ Correlations of miRNA quantity and flow cytometry parameters with *p* < 0.1 are reported.

### 3.5 Selected microRNAs are Risk Factors for PFS and OS in Multiple Myeloma and Plasma Cell Leukemia

The Cox proportional hazards model assessed risk factors associated with PFS and OS. The main risk factors for PFS of MM patients included low levels of miR‐143‐3p (*p* = 0.0412, cut‐off: 0.2848, Supporting Information S1: Figure S3A). In the case of OS, decreased expression of miR‐143‐3p (*p* = 0.0103, cut‐off: 0.1397, Supporting Information S1: Figure S3B) was found as a risk factor. In the case of PCL, low levels of miR‐191‐5p (*p =* 0.0042, cut‐off: 0.0965, Supporting Information S1: Figure S4A), miR‐744‐5p (*p* = 0.0344, cut‐off: 0.0544, Supporting Information S1: Figure S4B) were associated with shorter OS of PCL patients.

## 4 Discussion

Multiple myeloma is a hematological malignancy characterized by the uncontrolled proliferation of malignant PCs in the BM. The rapid disease progression into EMD or PCL is associated with increased numbers of circulating PCs in peripheral blood that are not dependent on growth signals from BM. The exact molecular mechanism leading to progression has yet to be fully elucidated. Nevertheless, numerous studies showed that non‐coding RNAs (including miRNA) are significantly dysregulated in MM and thus related to disease pathology, including tumor initiation, progression, or drug resistance [36, 37, 38]. They are present in small EVs and could be critical mediators in MM progression beyond the BM microenvironment [21].

In this study, we assessed the expression profiles of miRNAs isolated from small EVs present in BM plasma samples of MM, EMD, and PCL patients to identify new molecules responsible for MM progression. Using small RNA‐seq, we found 42 miRNAs differentially expressed among the analyzed subgroups. Interestingly, most were upregulated in MM patients, and their levels gradually declined through EMD to PCL progression. Further validation confirmed significant overexpression of miR‐140‐3p, and miR‐191‐5p in MM patients compared to the other two diagnoses. In addition, decreased levels of miR‐584‐5p and miR‐143‐3p in EMD compared to MM patients were detected.

Previously, a significant correlation between miR‐140‐3p expression and the recurrence of loss of heterozygosity (LOH) at 16q22.1‐q23.1 in MM patients was described [39]. The LOH at 16q22.1 correlated with the expression of *WWP2*, a host gene of miR‐140‐3p involved in ubiquitination processes [40]. Further, TNF‐α in the BM microenvironment contributes to the upregulation of this miRNA that, in turn, represses translation of DNMT1 and re‐expression of RANKL [41], a key molecule in bone remodeling and immune system development [42]. Recently, miR‐140‐3p was found to directly bind basic leucine zipper and W2 domain 2 (BZW2), a transcription factor possibly involved in the progression of MM. Importantly, downregulation of this miRNA led to increased proliferation, disrupted apoptosis, and impeded tumorigenesis of MM cell lines in nude mice [43]. In this study, low levels of miR‐140‐3p were detected in patients with significantly shorter OS. These results indicate a gradual decrease in miR‐140‐3p levels may be associated with MM progression into EMD or PCL from MM and shorter patient survival.

miR‐143‐3p functions as an important tumor suppressor in various cancers [44, 45]. Its involvement in MM pathogenesis has not been described, but similarly to miR‐140‐3p, we observed decreased levels of this miRNA in patients with shorter OS and PFS.

Concerning miR‐191‐5p and miR‐584‐5p, this is the first study demonstrating dysregulated levels of these miRNAs in MM compared to EMD or PCL patients. In colorectal cancer, expression of miR‐191‐5p was negatively correlated with PD‐L1; overexpression of this ligand was associated with poor survival and tumor recurrence [46]. Significantly, PD‐L1 is induced by the MM microenvironment; its elevated expression on PCs correlated with more proliferative potential, resistance to therapy, and disease progression due to the activation of AKT signaling [47]. This is in accordance with our results confirming significantly lower levels of miR‐191‐5p in patients with shorter OS and worse prognosis.

miR‐584‐5p is a crucial regulator of progression in many cancers, including neuroblastoma [48], non‐small cell lung cancer [49, 50], or hepatocellular carcinoma [51]. However, its role in hematological malignancies is not yet clarified. Recently, this miRNA was described to inhibit proliferation, migration, and invasion and promote apoptosis in osteosarcoma by targeting connective tissue growth factor (CTGF) [52]. Previous studies confirmed abnormally high levels of CTGF in MM patients compared to healthy controls; its elevated expression was observed in patients with osteolytic lesions and disease progression [53].

Finally, the dysregulation of miR‐744‐5p among the analyzed subgroups was not confirmed. Nevertheless, its low levels were linked to a shorter survival of patients. Our previous study found this miRNA to be significantly dysregulated in MM patients compared to the patients with monoclonal gammopathy of undetermined significance (MGUS) and healthy controls; its low levels were associated with disease progression and shorter OS [31]. Similarly, a decreased level of circulating miR‐744‐5p contained in small EVs isolated from serum samples of MM patients was considered a risk factor for progression and mortality [54]. Downregulation of this miRNA was previously confirmed as an independent predictor of poor prognosis in hepatocellular carcinoma after liver transplantation [55].

Furthermore, the correlation of flow cytometric parameters and miRNA expression levels suggests that miR‐584‐5p might be associated with increased aggressiveness, as evidenced by a higher number of CD138^+^CD38^+^ PCs, which are dominantly clonal. These results may reflect a shift toward a more malignant PC phenotype. A positive correlation was also observed for the expression of miR‐191‐5p and CD138^+^CD38^+^ PCs. The expression of both miR‐191‐5p and miR‐744‐5p was negatively correlated with nestin^+^ PCs. As previously reported, nestin^+^ PCs increase with disease progression [56]. Therefore, lower levels of these miRNAs may also be connected to increased aggressiveness.

To the best of our knowledge, this is the first study analyzing the expression profiles of small EV‐enriched miRNAs from BM plasma of MM patients and its aggressive variants EMD and PCL. Our results indicate that the dysregulation of miRNAs identified in this study contributes to MM progression into EMD or PCL. However, there is a critical need for advancements in technologies to characterize better the cargo of EV subpopulations, which would enhance our understanding and expand the diagnostic and therapeutic potential of EVs in cancers, neurodegenerative diseases, and infectious diseases [57].

## Author Contributions

JG, MVl, and SSe designed the research; JG, MVl, AD, JM, SSl, and MH performed the experiments; JG, MVl, PVF, LRa, JK, MJ, KB, and JJ analyzed the data; LRi, RB, MA, MS, IB, and LP provided clinical data and flowcytometric analyses; JG, MVl, and PVF wrote the article; JG, MVl, PVF, AD, TR, MVe, LRa, VP, SSl, MH, JK, MJ, JM, KB, JJ, LRi, RB, MA, IB, MS, LP, SSe reviewed and edited the article; SSe performed the revisions and final corrections. All authors approved the final version of the article.

## Ethics Statement

The study was approved by the University Hospital Brno Ethics Committee.

## Consent

All patients included in this study signed an informed consent form approved by the hospital's Ethics committee in accordance with the current Declaration of Helsinki.

## Conflicts of Interest

The authors declare no conflicts of interest.

### Peer Review

The peer review history for this article is available at https://www.webofscience.com/api/gateway/wos/peer-review/10.1002/hon.70036.

## Supporting information

Supporting Information S1

Supporting Information S2

## Data Availability

Small RNA sequencing data are accessible at https://www.ncbi.nlm.nih.gov/geo/query/acc.cgi?acc=GSE262424, access code ynozcosyzvmbpkt. The data will become publicly available after publication.
