# Supplementary material for: MicroRNA Profiling of Bone Marrow Plasma Extracellular Vesicles in Multiple Myeloma, Extramedullary Disease, and Plasma Cell Leukemia
Source: Hematol Oncol. 2025 Jan 13;43(1):e70036. doi: 10.1002/hon.70036 (PMC11727818; doi:10.1002/hon.70036)
Supplement: Supplementary file 1 — Supporting Information S1 [file HON-43-e70036-s001.docx]

**Supporting Information (Figures)


Figures legends:**

**Figure 1.** Characterization of small EVs from BM plasma samples. (a) MADLS measurements of EVs. Each graph shows average of six separate measurements for the sample. (a‘) Size and polydispersity index are reported as mean ± standard deviation (SD), concentration as mean (n = 6). (b) WB analysis for EVs markers/EV-associated proteins and a ‘negative marker’/non-EV associated protein ApoA-I.

**Figure 2:** A) Progression-free survival from multiple myeloma (MM), extramedullary disease (EMD) or plasma cell leukemia (PCL) diagnosis to progression, relapse, or all-cause death, whichever occurred first, B) Overall survival from MM/EMD/PCL diagnosis to all-cause death.

**Figure 3:** A) Low levels of miR-143-3p are associated with shorter progression-free survival of multiple myeloma (MM) patients (P = 0.0412, cut-off: 0.2848). B) Decreased levels of miR-143-3p are associated with shorter overall survival of MM patients (P = 0.0103, cut-off: 0.1397).

**Figure 4:** A) Low levels of miR-191-5p are associated with shorter overall survival of plasma cell leukemia (PCL) patients (P = 0.0042, cut-off: 0.0965). B) Decreased levels of miR-744-5p are associated with shorter overall survival of PCL patients (P = 0.0344, cut-off: 0.0544).


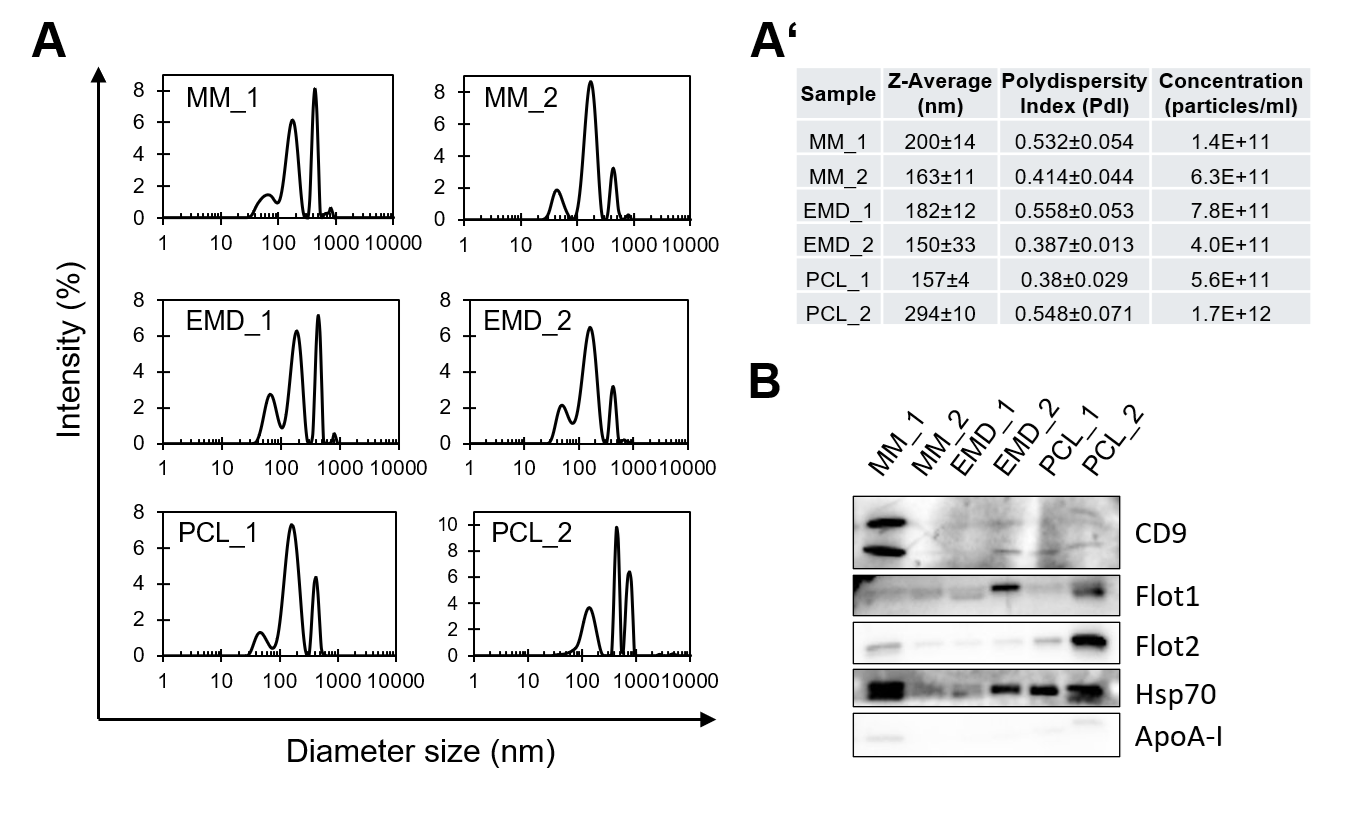


**Figure 1.** Characterization of small EVs from BM plasma samples. (a) MADLS measurements of EVs. Each graph shows average of six separate measurements for the sample. (a‘) Size and polydispersity index are reported as mean ± standard deviation (SD), concentration as mean (n = 6). (b) WB analysis for EVs markers/EV-associated proteins and a ‘negative marker’/non-EV associated protein ApoA-I.


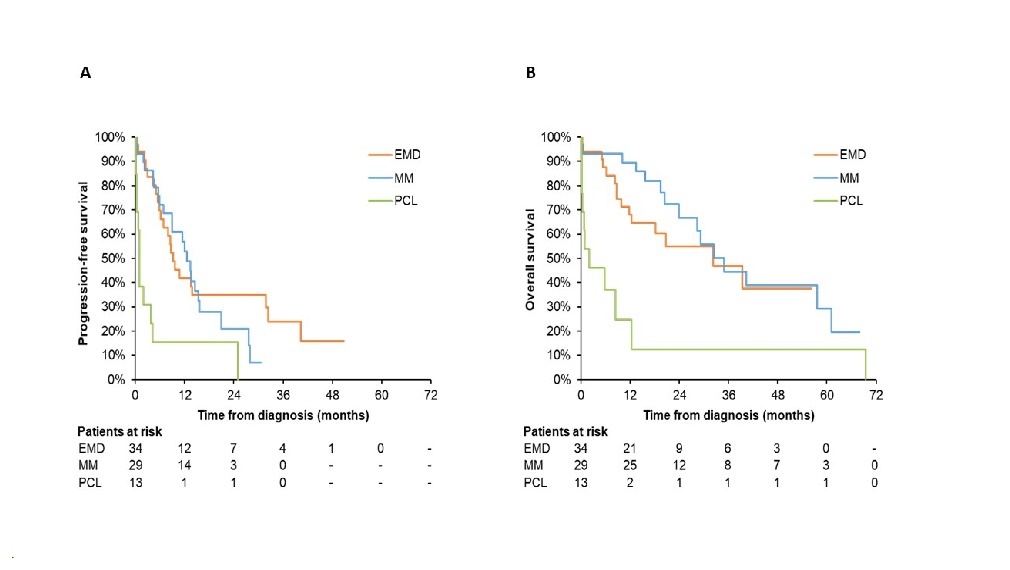


**Figure 2:** A) Progression-free survival from multiple myeloma (MM), extramedullary disease (EMD) or plasma cell leukemia (PCL) diagnosis to progression, relapse, or all-cause death, whichever occurred first, B) Overall survival from MM/EMD/PCL diagnosis to all-cause death.


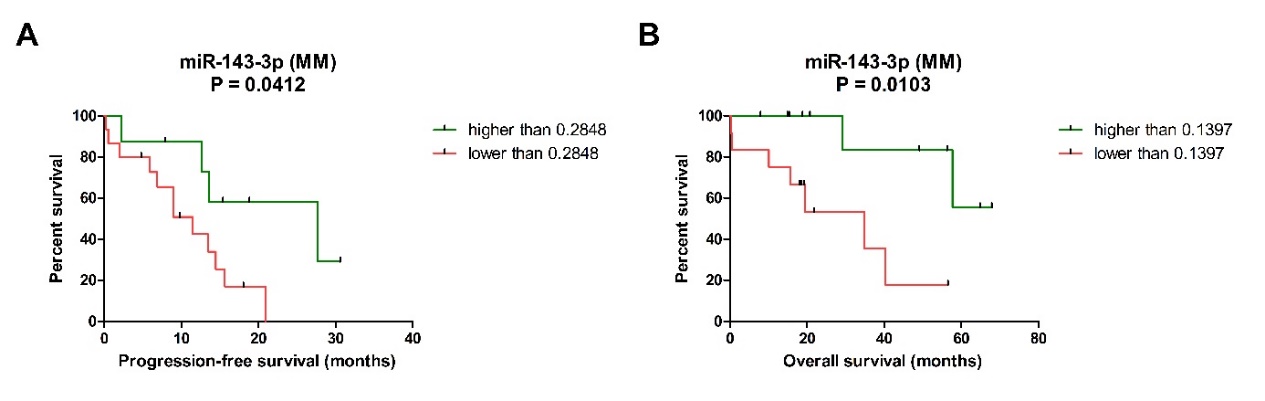


**Figure 3:** A) Low levels of miR-143-3p are associated with shorter progression-free survival of multiple myeloma (MM) patients (P = 0.0412, cut-off: 0.2848). B) Decreased levels of miR-143-3p are associated with shorter overall survival of MM patients (P = 0.0103, cut-off: 0.1397).


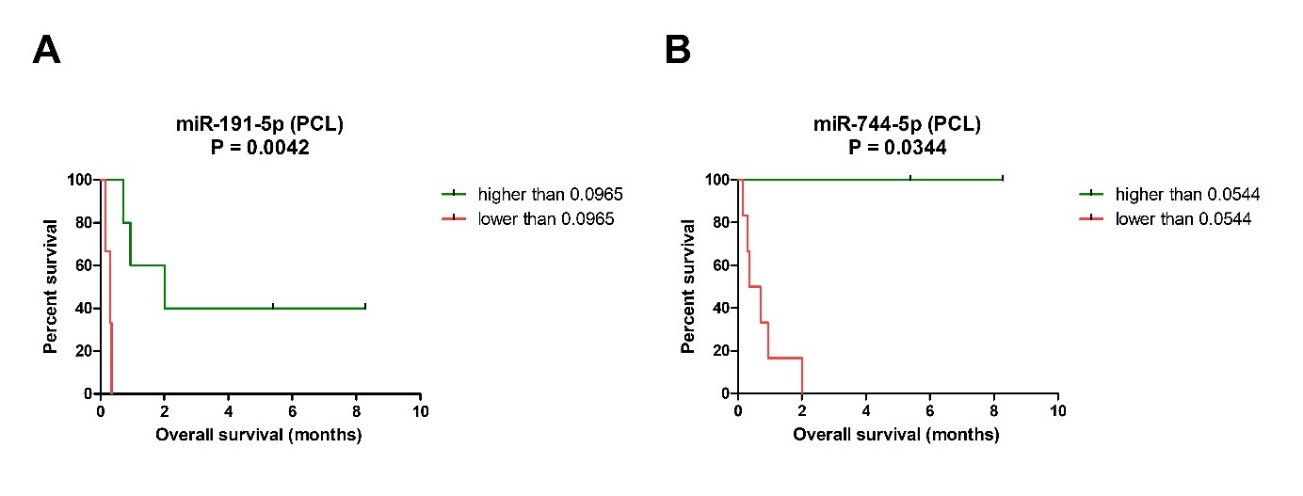


**Figure 4:** A) Low levels of miR-191-5p are associated with shorter overall survival of plasma cell leukemia (PCL) patients (P = 0.0042, cut-off: 0.0965). B) Decreased levels of miR-744-5p are associated with shorter overall survival of PCL patients (P = 0.0344, cut-off: 0.0544).
