# Supplementary material for: MicroRNA Profiling of Bone Marrow Plasma Extracellular Vesicles in Multiple Myeloma, Extramedullary Disease, and Plasma Cell Leukemia
Source: Hematol Oncol. 2025 Jan 13;43(1):e70036. doi: 10.1002/hon.70036 (PMC11727818; doi:10.1002/hon.70036)
Supplement: Supplementary file 2 — Supporting Information S2 [file HON-43-e70036-s002.docx]

**Supplementary Table S1:** Baseline clinical characteristics of patients

| **Parameters**^1^ |  | **MM (N = 29)** | **EMD (N = 34)** | **PCL (N = 13)** |
| --- | --- | --- | --- | --- |
| Sex | Man | 16 (55.2 %) | 24 (70.6 %) | 7 (53.8 %) |
|  | Woman | 13 (44.8 %) | 10 (29.4 %) | 6 (46.2 %) |
| Age | Median (min–max) | 69 (44–82) | 62 (44–82) | 66 (31–82) |
| ECOG | 0 | 2 (6.9 %) | 9 (26.5 %) | 1 (7.7 %) |
|  | 1 | 15 (51.7 %) | 9 (26.5 %) | 7 (53.8 %) |
|  | 2 | 9 (31.0 %) | 13 (38.2 %) | 5 (38.5 %) |
|  | 3 | 2 (6.9 %) | 3 (8.8 %) | 0 (0.0 %) |
|  | 4 | 1 (3.4 %) | 0 (0.0 %) | 0 (0.0 %) |
| ISS | Stage 1 | 6 (20.7 %)^a^ | 19 (55.9 %) | 2 (15.4 %)^a^ |
|  | Stage 2 | 6 (20.7 %) | 6 (17.6 %) | 2 (15.4 %) |
|  | Stage 3 | 17 (58.6 %) | 9 (26.5 %) | 9 (69.2 %) |
| Durie-Salmon stage | I | 2 (6.9 %)^a^ | 0 (0.0 %) | 0 (0.0 %)^a^ |
|  | II | 6 (20.7 %) | 1 (2.9 %) | 6 (46.2 %) |
|  | III | 21 (72.4 %) | 33 (97.1 %) | 7 (53.8 %) |
| Durie-Salmon substage | A | 21 (72.4 %)^a^ | 29 (85.3 %)^a^ | 4 (30.8 %) |
|  | B | 8 (27.6 %) | 5 (14.7 %) | 9 (69.2 %) |
| M-protein type | Biclonal | 0 (0.0 %) | 1 (2.9 %) | 0 (0.0 %) |
|  | IgA | 7 (24.1 %) | 12 (35.3 %) | 1 (7.7 %) |
|  | IgG | 20 (69.0 %) | 16 (47.1 %) | 7 (53.8 %) |
|  | IgM | 0 (0.0 %) | 0 (0.0 %) | 1 (7.7 %) |
|  | LC only | 2 (6.9 %) | 3 (8.8 %) | 3 (23.1 %) |
|  | Nonsecretory | 0 (0.0 %) | 2 (5.9 %) | 1 (7.7 %) |
| Serum M-protein quantity (g·l^-1^) | Median (min–max) | 43.8 (1.5–78.2)^b^ | 19.3 (0.0–80.8)^a^ | 25.4 (0.0–80.2)^ab^ |
| Light chain type | Biclonal | 0 (0.0 %) | 1 (2.9 %) | 0 (0.0 %) |
|  | Kappa | 22 (75.9 %) | 15 (44.1 %) | 5 (38.5 %) |
|  | Lambda | 7 (24.1 %) | 16 (47.1 %) | 7 (53.8 %) |
|  | Unknown | 0 (0.0 %) | 2 (5.9 %) | 1 (7.7 %) |
| Hemoglobin level  (g·l^-1^) | Median (min–max) | 100 (54–131)^a^ | 111 (72–158) | 91 (69–149)^a^ |
| Thrombocyte count (10^9^·l^-1^) | Median (min–max) | 194 (89–366) | 229 (29–480) | 180 (63–273) |
| Calcium total level (mmol·l^-1^) | Median (min–max) | 2.4 (1.8–3.7) | 2.4 (1.9–3.7) | 2.5 (2.0–4.7) |
| Albumin level (g·l^-1^) | Median (min–max) | 34.0 (19.0–48.4)^b^ | 39.0 (25.0–48.9)^a^ | 35.1 (22.5–49.8)^ab^ |
| Creatinine level (µmol·l^-1^) | Median (min–max) | 99 (55–1 138)^ab^ | 85 (33–772)^a^ | 195 (66–1 208)^b^ |
| β_2_-microglobulin (mg·l^-1^) | Median (min–max) | 5.7 (0.1–50.0)^a^ | 3.5 (1.3–28.9) | 6.9 (2.2–44.9)^a^ |
| Lactate dehydrogenase (µkat·l^-1^) | Median (min–max) | 2.6 (1.4–6.6) | 3.5 (1.5–24.5) | 5.5 (2.7–9.0) |
| C-reactive protein (mg·l^-1^) | Median (min–max) | 6.2 (0.0–97.9) | 5.1 (0.0–118.5) | 7.9 (0.0–136.0) |
| Plasmocyte count (%) – bone marrow cytology | Median (min–max) | 21.0 (4.8–66.4) | 15.5 (0.0–70.4) | 53.2 (15.0–85.8) |

MM – multiple myeloma, EMD – extramedullary disease, PCL – plasma cell leukemia

*^1^* Described using n (%) in categorical variables and median (minimum–maximum) in continuous variables.

**Supplementary Table S2:** Fluorescently labelled monoclonal antibodies used for flow cytometry

| **Antibody** | **Clone** | **Supplier** |
| --- | --- | --- |
| CD19-PC7 | J3-119 | Beckman Coulter |
| CD20-FITC | 2H7 | Exbio |
| CD27-APC-A750 | 1A4CD27 | Beckman Coulter |
| CD28-FITC | CD28.2 | Exbio |
| CD38-PB | HIT2 | Exbio |
| CD44-APC-eFluor780 | IM7 | eBioscience |
| CD56-PE/APC | LT56 | Exbio |
| CD81-APC-H7 | JS-81 | Becton Dickinson |
| CD117-APC | 104D2 | Exbio |
| CD138-PerCP | MI15 | Exbio |
| CD200-PE | OX-104 | Exbio |
| Nestin-APC | 196908 | R&D |

**Supplementary Table S3:** TaqMan Advanced microRNA Assays used in the validation phase of the study.

| **miRNA** | **Assay ID** |
| --- | --- |
| hsa-miR-744-5p | 478200_mir |
| hsa-miR-143-3p | 477912_mir |
| hsa-miR-140-3p | 477908_mir |
| hsa-miR-584-5p | 478167_mir |
| hsa-miR-191-5p | 477952_mir |
| hsa-miR-26a-5p | 477995_mir |

**Supplementary table S4:** Comparison of cytogenetic data in multiple myeloma (MM), extramedullary disease (EMD), and plasma cell leukemia (PCL) patients.

| **Parameters** | **Status** | **MM (N = 29)** | **EMD (N = 34)** | **PCL (N = 13)** | ***P*-value** |
| --- | --- | --- | --- | --- | --- |
| Cytogenetics | No | 9 (31.0 %) | 10 (29.4 %) | 3 (23.1 %) | 0.893 |
|  | Yes | 20 (69.0 %) | 24 (70.6 %) | 10 (76.9 %) |  |
| FISH | Not evaluable | 3 (15.0 %) | 7 (29.2 %) | 0 (0.0 %) | 0.124 |
|  | Yes | 17 (85.0 %) | 17 (70.8 %) | 10 (100.0 %) |  |
| IgH disruption | Negative | 9 (52.9 %) | 14 (82.4 %) | 1 (10.0 %) | **0.002** |
|  | Positive | 7 (41.2 %) | 3 (17.6 %) | 8 (80.0 %) |  |
|  | Unknown | 1 (5.9 %) | 0 (0.0 %) | 1 (10.0 %) |  |
| t(11;14) | Negative | 0 (0.0 %) | 4 (23.5 %) | 1 (10.0 %) | 0.524 |
|  | Positive | 1 (5.9 %) | 1 (5.9 %) | 0 (0.0 %) |  |
|  | Unknown | 16 (94.1 %) | 12 (70.6 %) | 9 (90.0 %) |  |
| t(4;14) | Negative | 9 (52.9 %) | 10 (58.8 %) | 4 (40.0 %) | **0.012** |
|  | Positive | 1 (5.9 %) | 0 (0.0 %) | 4 (40.0 %) |  |
|  | Unknown | 7 (41.2 %) | 7 (41.2 %) | 2 (20.0 %) |  |
| t(6;14) | Negative | 0 (0.0 %) | 4 (23.5 %) | 0 (0.0 %) | n.a. |
|  | Positive | 0 (0.0 %) | 0 (0.0 %) | 0 (0.0 %) |  |
|  | Unknown | 17 (100.0 %) | 13 (76.5 %) | 10 (100.0 %) |  |
| t(14;16) | Negative | 8 (47.1 %) | 7 (41.2 %) | 4 (40.0 %) | n.a. |
|  | Positive | 0 (0.0 %) | 0 (0.0 %) | 0 (0.0 %) |  |
|  | Unknown | 9 (52.9 %) | 10 (58.8 %) | 6 (60.0 %) |  |
| del(13)(q14)/monosomy 13 | Negative | 7 (41.2 %) | 7 (41.2 %) | 3 (30.0 %) | 0.852 |
|  | Positive | 10 (58.8 %) | 9 (52.9 %) | 7 (70.0 %) |  |
|  | Unknown | 0 (0.0 %) | 1 (5.9 %) | 0 (0.0 %) |  |
| gain 1q21 | Negative | 9 (52.9 %) | 6 (35.3 %) | 1 (10.0 %) | 0.087 |
|  | Positive | 8 (47.1 %) | 10 (58.8 %) | 9 (90.0 %) |  |
|  | Unknown | 0 (0.0 %) | 1 (5.9 %) | 0 (0.0 %) |  |
| del(17)(p13) | Negative | 14 (82.4 %) | 11 (64.7 %) | 8 (80.0 %) | 0.999 |
|  | Positive | 3 (17.6 %) | 3 (17.6 %) | 2 (20.0 %) |  |
|  | Unknown | 0 (0.0 %) | 3 (17.6 %) | 0 (0.0 %) |  |
| Hyperdiploidy | No | 3 (17.6 %) | 1 (5.9 %) | 4 (40.0 %) | 0.758 |
|  | Yes | 1 (5.9 %) | 1 (5.9 %) | 1 (10.0 %) |  |
|  | Unknown | 13 (76.5 %) | 15 (88.2 %) | 5 (50.0 %) |  |

FISH – fluorescent in-situ hybridization, n.a. - not available

**Supplementary Table S5:** Comparison of flow‑cytometry data in multiple myeloma (MM), extramedullary disease (EMD), and plasma cell leukemia (PCL) patients.

| **Parameter** | **n** | **MM (n = 29)**  **Median (min-max)** | **n** | **EMD (n = 34)**  **Median (min-max)** | **n** | **PCL (n = 13)**  **Median (min-max)** | ***P*-value^1^** |
| --- | --- | --- | --- | --- | --- | --- | --- |
| PCs CD138+CD38+ | 29 | 9.2 (0.1–52.6)^a^ | 34 | 4.4 (0.0–69.9)^a^ | 13 | 41.7 (11.0–85.9) | **< 0.001** |
| CD56+ PCs | 29 | 99.0 (0.0–100.0) | 31 | 98.5 (0.0–100.0) | 13 | 98.9 (0.0–99.9) | 0.940 |
| CD19+ PCs | 29 | 0.1 (0.0–81.0) | 31 | 0.0 (0.0–70.8)^a^ | 13 | 0.0 (0.0–0.8)^a^ | **0.013** |
| CD20+ PCs | 29 | 0.1 (0.0–66.4) | 31 | 0.1 (0.0–99.4) | 13 | 0.0 (0.0–92.8) | 0.526 |
| CD27+ PCs | 29 | 3.1 (0.0–95.9) | 31 | 3.7 (0.0–97.7) | 13 | 0.4 (0.0–90.1) | 0.154 |
| CD28+ PCs | 27 | 0.0 (0.0–98.8)^a^ | 28 | 0.2 (0.0–100.0)^ab^ | 13 | 0.1 (0.0–99.5)^b^ | **0.047** |
| CD44+ PCs | 27 | 89.3 (1.0–100.0) | 27 | 89.7 (3.7–100.0) | 9 | 85.4 (3.0–100.0) | 0.668 |
| CD81+ PCs | 27 | 4.4 (0.0–98.5) | 28 | 4.3 (0.0–95.1) | 10 | 1.2 (0.3–96.2) | 0.286 |
| CD117+ PCs | 28 | 38.4 (0.0–98.4)^a^ | 28 | 7.4 (0.0–99.5)^ab^ | 13 | 0.1 (0.0–92.4)^b^ | **0.039** |
| nestin+ PCs | 28 | 0.4 (0.0–96.4) | 25 | 0.4 (0.0–100.0) | 9 | 2.4 (0.0–99.5) | 0.306 |
| CD200+ PCs | 23 | 98.6 (0.0–980.0)^b^ | 24 | 63.8 (0.0–99.6)^a^ | 5 | 34.1 (0.3–99.9)^ab^ | **0.045** |
| polyclonal PCs | 29 | 0.6 (0.0–6.6) | 34 | 3.3 (0.0–100.0) | 13 | 0.0 (0.0–0.1) | **< 0.001** |
| clonal-PCs | 29 | 99.4 (93.4–100.0) | 34 | 96.7 (0.0–100.0) | 13 | 100.0 (99.9–100.0) | **< 0.001** |

^1^ *P*-value of Kruskal-Wallis test. PC – plasma cells

^a, b^, Same letters denote groups of patients that do not significantly differ (post hoc analysis).

**Supplementary Table S6:** Correlation of microRNA expression levels with continuous clinical characteristics of patients at diagnosis.

| **miRNA** | **Clinical characteristic** | **N** | ***r*_S_** | ***P*-value^1^** |
| --- | --- | --- | --- | --- |
| miR-140-3p | LDH (µkat·l^-1^) | 63 | -0.360 | **0.004** |
| miR-584-5p | Serum M-protein quantity (g·l^-1^) | 58 | 0.247 | 0.062 |
|  | Hemoglobin level (g·l^-1^) | 64 | -0.404 | **0.001** |
|  | Albumin level (g·l^-1^) | 64 | -0.330 | **0.008** |
|  | Creatinine level (µmol·l^-1^) | 64 | 0.243 | 0.053 |
|  | β-2-microglobulin (mg·l^-1^) | 63 | 0.222 | 0.081 |
|  | LDH (µkat·l^-1^) | 63 | -0.241 | 0.057 |
|  | Plasmocyte count (%) – bone marrow cytology | 64 | 0.271 | **0.030** |
| miR-191-5p | Age | 64 | -0.286 | **0.022** |
|  | Serum M-protein quantity (g·l^-1^) | 58 | 0.339 | **0.009** |
|  | LDH (µkat·l^-1^) | 63 | -0.369 | **0.003** |
|  | CRP (mg·l^-1^) | 64 | -0.291 | **0.020** |
| miR-143-3p | Serum M-protein quantity (g·l^-1^) | 58 | 0.441 | **0.001** |
|  | LDH (µkat·l^-1^) | 63 | -0.397 | **0.001** |
|  | Plasmocyte count (%) – bone marrow cytology | 64 | 0.211 | 0.094 |
| miR-744-5p | LDH (µkat·l^-1^) | 63 | -0.270 | **0.032** |

^1^ Correlations of miRNA quantity and clinical characteristics with *P* < 0.1 are reported.
